# Young-Adult Male Rats' Vulnerability to Chronic Mild Stress Is Reflected by Anxious-Like instead of Depressive-Like Behaviors

**DOI:** 10.1155/2016/5317242

**Published:** 2016-06-28

**Authors:** Herrera-Pérez José Jaime, Benítez-Coronel Venus, Jiménez-Rubio Graciela, Hernández-Hernández Olivia Tania, Martínez-Mota Lucía

**Affiliations:** ^1^Laboratorio de Farmacología Conductual, Dirección de Investigaciones en Neurociencias, Instituto Nacional de Psiquiatría Ramón de la Fuente Muñiz, Calzada México-Xochimilco 101, Colonia San Lorenzo Huipulco, Delegación Tlalpan, 14370 Ciudad de México, DF, Mexico; ^2^Consejo Nacional de Ciencia y Tecnología, Instituto Nacional de Psiquiatría Ramón de la Fuente Muñiz, Calzada México-Xochimilco 101, Colonia San Lorenzo Huipulco, Delegación Tlalpan, 14370 Ciudad de México, DF, Mexico

## Abstract

In a previous study, we found that chronic mild stress (CMS) paradigm did not induce anhedonia in young-adult male rats but it reduced their body weight gain. These contrasting results encouraged us to explore other indicators of animal's vulnerability to stress such as anxious-like behaviors, since stress is an etiologic factor also for anxiety. Thus, in this study, we evaluated the vulnerability of these animals to CMS using behavioral tests of depression or anxiety and measuring serum corticosterone. Male Wistar rats were exposed to four weeks of CMS; the animals' body weight and sucrose preference (indicator of anhedonia) were assessed after three weeks, and, after the fourth week, some animals were evaluated in a behavioral battery (elevated plus maze, defensive burying behavior, and forced swimming tests); meanwhile, others were used to measure serum corticosterone. We found that CMS (1) did not affect sucrose preference, immobility behavior in the forced swimming test, or serum corticosterone; (2) decreased body weight gain; and (3) increased the rat's entries into closed arms of the plus maze and the cumulative burying behavior. These data indicate that young male rats' vulnerability to CMS is reflected as poor body weight gain and anxious-like instead of depressive-like behaviors.

## 1. Introduction

According to Seyle [1, 2], stress is defined as the nonspecific response of the body to any demand. This response is induced by any new situation (chemical, physical, environmental, emotional, and psychosocial stressor) which disturbs the homeostasis and induces a general adaptive response aimed at restoring the initial level of stability (adaptation). All the manifestations of the adaptive response are beneficial to the organism when limited in time, but when the duration of the stress is excessive it contributes to the development of pathological conditions [1, 2]. Thus, the diathesis-stress hypothesis of affective disorders states that chronic stress could induce physiological alterations that promote an exaggerated stress response, which in turn would cause depressive or anxious events [3, 4].

Depression is a mood disorder characterized by anhedonia (incapacity to experience pleasure), feelings of sadness and guilt, depressed mood, hopelessness, and suicidal thoughts [5]; meanwhile, anxiety is distinguished by excessive and inappropriate worrying (i.e., persistent and not restricted to particular circumstances), restlessness, difficulty concentrating, irritability, muscle tension, and disturbed sleep [5]. To study these pathologies, researchers have used animal models to simulate a symptom of the human disease [6]; most of these models are based on rodent exposure to a stressor. Chronic mild stress (CMS) is an animal model that simulates anhedonia; in this paradigm, constant exposure of the animal to several stressors of moderate intensity during a period of several weeks leads to a reduction of the consumption of a palatable sucrose solution, indicating an anhedonic state [7]. It has been established that this model has high validity (face, predictive, and construct validity) that makes it a useful tool to study the neurobiology of depression [8]. In agreement with the diathesis-stress hypothesis of affective disorders, several studies have found that rodent's exposure to CMS can induce several behavioral alterations (different from anhedonia) that mimic human symptoms of depression [8–10] and anxiety [11, 12]. Recently, using this model, we found that a high proportion of young-adult male rats did not develop anhedonia when exposed to a CMS paradigm, suggesting that these animals are resilient to this kind of stress [13]. Contrary to this, we also found that the CMS reduced the rats' body weight gain as compared to unstressed rats [13]. These data indicate that the chronic stress schedule affects the physiology of the animal, but this is not enough to induce an anhedonic state. These results encourage finding other indicators to evaluate the vulnerability of young-adult rats to CMS; this could be done by scanning a broader spectrum of behaviors or phenotypes potentially impaired or induced by this kind of stress and by measuring the activity of hypothalamic-pituitary-adrenal (HPA) axis.

On these bases, we hypothesize that the young rats that did not develop anhedonia when exposed to CMS present other behavioral alterations that suggest depression and/or anxiety. Thus, the objective of this study was to evaluate the vulnerability of young-adult rats to CMS using behavioral tests that detect depressive- or anxious-like behaviors and measuring corticosterone serum levels.

## 2. Materials and Methods

### 2.1. Animals

Young (3-month-old) male Wistar rats were obtained from the vivarium of the* Instituto Nacional de Psiquiatría Ramón de la Fuente Muñiz* (INPRFM). Animals were individually housed in cages measuring 27 × 16 × 23 cm and maintained on an inverted 12 h dark-light cycle condition (lights off at 10:00 a.m.), under controlled temperature and humidity. The animals had free access to water and food, except for the periods required by the CMS procedure. Animal management was done according to the general principles of laboratory animal care [14]. All experimental procedures were performed in accordance with the Mexican official norm for animal care and handling [15] and approved by the Ethical Committee of the INPRFM. All efforts were made to minimize the number of animals used and their suffering.

### 2.2. Chronic Mild Stress Model

#### 2.2.1. Sucrose Consumption Training

Animals were allowed to adapt to the taste of a palatable sucrose solution (1%) for two weeks. During this period, a bottle containing sucrose solution was presented to the rats daily for one hour.

#### 2.2.2. Baseline Sucrose Consumption

The baseline sucrose consumption was determined two days after the training period at the beginning of the dark phase (at 10:00 a.m.). For this purpose, the rats were water and food deprived for 20 h and thereafter presented with two bottles during a period of one hour: one containing sucrose solution (1%) and the other tap water. The objective of the food and water deprivation period is to stimulate the liquid consumption in the animals in order to have a good dynamic range to evaluate differences in fluid intake [16]. Fluid (sucrose solution or tap water) consumption was calculated by weighing the bottles before and after exposure to the animals. For baseline body weight measurements, animals were weighed immediately before water and food deprivation.

#### 2.2.3. Experimental Design for Behavioral Study

**Figure 1:**
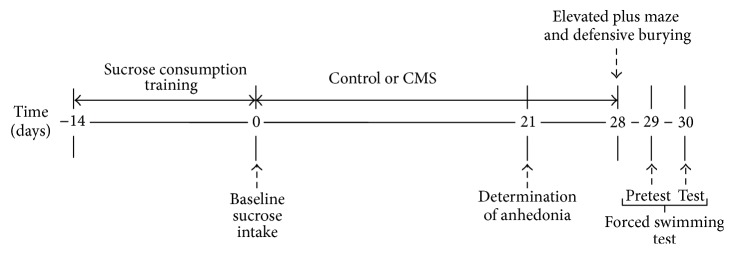
Timeline for experimental manipulations.

After determination of baseline sucrose consumption, young-adult male rats were randomly assigned to a control or stress group. The males in the control group (*n* = 13) were maintained during a period of four weeks without stress, but under similar handling and storage conditions to the stressed animals. The stress group (*n* = 13) was exposed, during a period of four weeks, to several stressors: white noise (~90 dB), overcrowding (2-3 animals per cage), continuous lighting, soiled cage (250 mL water spilled into bedding), stroboscopic light (300 flashes/min), 45° cage tilt along the vertical axis, and water deprivation. The stressor schedule followed in this study was previously used [13, 17, 18] and it is shown in Table 1. To determine anhedonia development, we used a within-subject design since the effect of stress on hedonic state is progressive and the kinetics of the changes in fluid intake are important to determine the establishment of anhedonia in the animals. Thus, during the first three weeks of CMS, sucrose and water intake was determined weekly, as indicated below. At the fourth week (day 27), the 20 h of water and food deprivation indicated in Table 1 was omitted and at this time the CMS was finished. Afterwards, a behavioral tests battery was performed to evaluate stress vulnerability beyond anhedonia; we used a between-subject design in order to avoid ceiling effects or reduction of exploration originated by repeated animals' exposure to the tests. Then, on day 28, the control and stressed groups were exposed to the elevated plus maze test and, immediately after, to the defensive burying behavior test. On days 29 and 30, animals were evaluated in the forced swimming test (pretest and test, resp., at 2:00 p.m.). Several studies indicate that animal's performance in these behavioral tests is sensible to previous stress exposure, including CMS [19–22]; thus, they could be good indicators of stress vulnerability in the nonanhedonic rats. The tests are described below and the time flow of experimental manipulations is indicated in Figure 1.

### 2.3. Sucrose Preference Test and Body Weight Measurement

In control and stressed animals, the sucrose solution and tap water consumption were determined weekly as indicated in the baseline sucrose consumption section. These measurements (for the control and stressed animals) were done at 10:00 a.m., after a 20 h period of water and food deprivation. Rat's body weight was measured weekly, immediately before the 20 h of water and food deprivation. To avoid changes determined by baseline differences, individual sucrose and water consumption were expressed as relative sucrose or water intake; this parameter was calculated by dividing the sucrose or water consumption at any time by the respective baseline ingestion; this parameter has statistical sensibility to reductions in sucrose solution intake [18]. Sucrose preference was calculated weekly by dividing the sucrose intake by total fluid (sucrose plus water) consumption and the result was expressed as percentage. Finally, rat's body weight was expressed as a percentage relative to baseline body weight in order to discard effects of baseline differences.

The anhedonic state generated by the exposure to CMS schedule would be indicated by a reduction in the weekly sucrose consumption or preference after three weeks of stress [7, 13, 18].

### 2.4. Elevated Plus Maze Test

The elevated plus maze is an animal model of anxiety based on rodents' aversion for open spaces [23]. The test setting consisted of a plus-shaped apparatus (arms size: 50 × 10 cm) placed 50 cm above the floor with two open and two closed arms (enclosed by 40 cm high walls). The experiments were performed under dim red light. The rats were placed at the intersection of the four arms of the maze facing an open arm. The test was videotaped during a period of 10 min for posterior analysis. The maze was cleaned after each testing. The number of entries and the time spent in the closed or open arms were measured. An increment of rat's anxiety-like state induced by CMS would be indicated by increasing the time spent or the number of entries in the closed arms [24].

### 2.5. Defensive Burying Behavior Test

The burying behavior test was used to assess experimental anxiety and it is based on the rat's innate behavior to bury aversive stimuli [25]. The test was performed in a cage measuring 27 × 16 × 23 cm with an electrified probe (7 cm long, 2 cm above the bedding material) placed in one of the cage walls. When the rat touched the electrified probe with its snout or forepaws, it received a 0.3 mA shock. The experiments were performed under dim red light. The rats were exposed to this cage during a period of 10 min, and the test was videotaped for posterior analysis. The number of prod shocks, the burying behavior latency (time between the first shock and the display of burying behavior), and the cumulative burying behavior (total amount of time spent spraying the bedding material towards and on top of the shock probe) were measured. An anxiogenic effect of CMS would be indicated by a decrease of the burying behavior latency and/or an increase of the cumulative burying behavior.

### 2.6. Forced Swimming Test

The forced swimming test (FST) is a commonly used animal model of depression that evaluates behavioral despair. In this study, we used the modified FST [26]. Swimming sessions were conducted by placing the rat in a glass cylinder (46 cm tall × 20 cm in diameter) containing water (30 cm depth) at 23–25°C. The FST consisted of two swimming sessions 24 h apart. In the first session (pretest), rats were allowed to swim for 15 min. Twenty-four hours later, animals were subjected to a 5 min swimming session (test). At the end of each session, rats were removed from the jar, dried with paper towel, and placed in a cage for 15 min before returning them to the home cages. The test was videotaped and a time-sampling technique was used to score, every 5 s, the presence of immobility behavior (floating without struggling and making only those movements necessary to keep the head above the water). Behavioral despair would be indicated by high levels of immobility.

### 2.7. Corticosterone Serum Measurements

To evaluate the effect of CMS on HPA activity, we measured corticosterone serum levels. It has been demonstrated that rats' exposure to behavioral tests such as the FST could increase their HPA axis activity [27]; this effect could veil the impact of the CMS protocol on the function of this axis. Thus, in the current study, the corticosterone levels were measured in two independent groups of rats that were experimentally manipulated as indicated above (see Figure 1), except that they were not exposed to the elevated plus maze, the defensive behavior, or the forced swimming tests. During a period of four weeks, one of these groups was exposed to CMS (*n* = 7); meanwhile, the other was kept on control condition (*n* = 6); according to the sucrose preference, after three weeks of CMS, none of the stressed rats developed anhedonia (data not shown). On day 28, the animals of both groups were sacrificed by decapitation and their trunk blood was collected in cold tubes. The blood was centrifuged (4000 rpm for 25 min at 4°C) to obtain serum samples that were stored at −4°C until analysis. Corticosterone concentrations were measured by radioimmunoassay using a commercial kit (Siemens Rat Corticosterone Coat-A-Count Kit, TKRC1); this procedure used antibody-coated tubes in which ^125^I-labelled rat corticosterone competed with the free hormone in the sample for antibody sites. Corticosterone (ng/mL) total quantity was determined using a calibration curve. The interassay and intra-assay variabilities were 4.8 and 4.0%, respectively.

### 2.8. Statistics

Tap water or sucrose solution intake, sucrose preference, and relative body weight were analyzed by two-way repeated measures analysis of variance (RM ANOVA) including the factors stress exposure and time, followed by Tukey as the* post hoc* test. Student's *t*-test was used to analyze (a) the time spent and the number of entrances in the open or closed arm of the elevated plus maze; (b) the number of shocks, the burying behavior latency, or the cumulative burying behavior in the defensive burying behavior test; (c) the immobility in the forced swimming test; and (e) corticosterone serum levels. Statistical analysis was carried out using the Sigma Plot software, version 11. A value of *p* < 0.05 was considered statistically significant.

## 3. Results

### 3.1. Anhedonia Development in the CMS

**Figure 2:**
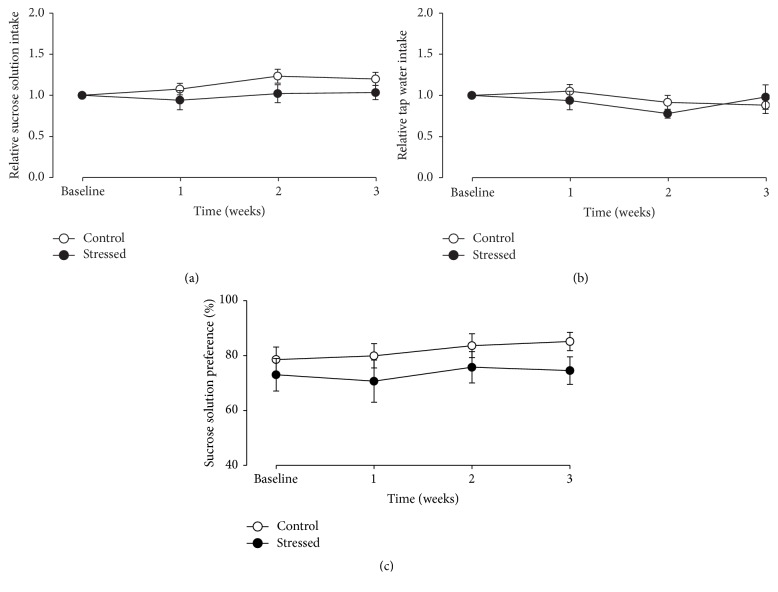
CMS failed to induce anhedonia in the animals. The sucrose solution (a) and tap water intake (b) as well as the sucrose solution preference (c) of rat exposed to CMS (black circles) or maintained without stress (white circles) did not change throughout the experiment. Fluid intake of sucrose solution and tap water was expressed as relative to basal consumption in order to avoid baseline differences. Data are expressed as mean ± SEM.

Figure 2 shows the sucrose intake (a), the tap water consumption (b), and the sucrose preference (c) of rats exposed to CMS (*n* = 13, black circles) or maintained without stress (*n* = 13, white circles). The two-way RM ANOVA indicated that sucrose solution intake was not affected by stress (*F*_1,24_ = 2.537, *p* = 0.124), time of exposure (*F*_3,72_ = 2.175, *p* = 0.098), or the interaction of both factors (*F*_3,72_ = 0.975, *p* = 0.409). In a similar way, tap water consumption did not show significant differences (RM ANOVA, stress: *F*_1,24_ = 0.172, *p* = 0.682; time of exposure: *F*_3,72_ = 2.151, *p* = 0.101; and interaction: *F*_3,72_ = 1.248, *p* = 0.299). Regarding sucrose preference, the statistics indicated differences determined by time (*F*_3,72_ = 3.011, *p* = 0.036) but not by stress (*F*_1,24_ = 1.385, *p* = 0.251) or the interaction of both factors (*F*_3,72_ = 0.584, *p* = 0.627); the main effect of time is due to increase of preference along the experiment (Figure 2(c)).

### 3.2. Relative Body Weight

**Figure 3:**
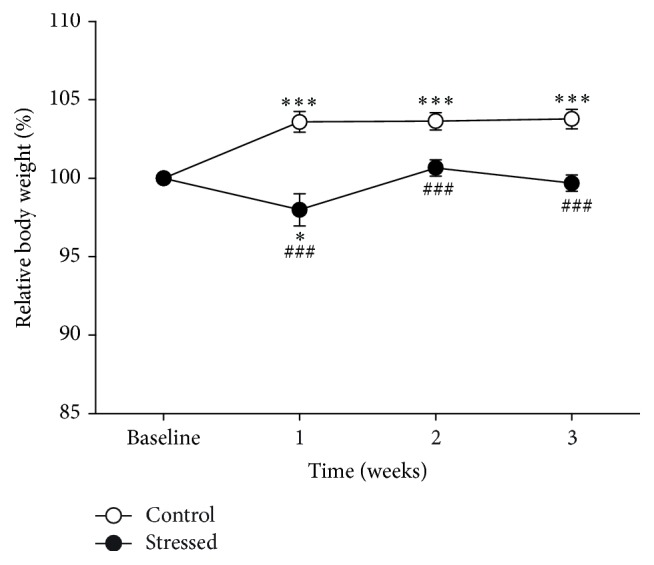
The rats' growing rate is reduced by CMS exposure. Compared to unstressed rats (white circles), the relative body weights of rats exposed to CMS (black circles) are lower at the three weeks of stress exposure. Values are expressed as percentage of baseline to avoid effects determined by baseline differences. Data are expressed as mean ± SEM. Tukey test: ^*∗*^ *p* < 0.05 and ^*∗∗∗*^ *p* < 0.001 versus baseline; ^###^ *p* < 0.001 versus control group.

Relative body weight of rats exposed to CMS (*n* = 13, black circles) or maintained without stress (*n* = 13, white circles) is shown in Figure 3. Data analysis indicates a significant effect of stress (*F*_1,24_ = 37.919, *p* < 0.001), time of exposure (*F*_3,72_ = 6.762, *p* < 0.001), and the interaction of both factors (*F*_3,72_ = 10.213, *p* < 0.001).* Post hoc* analysis indicated that the body weight of control rats at week 1, 2, or 3 is higher than their basal value (*p* < 0.001 in all cases); meanwhile, in the stressed group, only the body weight at week 1 was lower than their basal level (*p* = 0.041). Tukey test also evidenced the notion that body weight of the stressed group was lower than the weight in the control group at weeks 1, 2, and 3 (*p* < 0.001 in all cases).

### 3.3. Anxiety-Like Behavior in the Elevated Plus Maze

**Figure 4:**
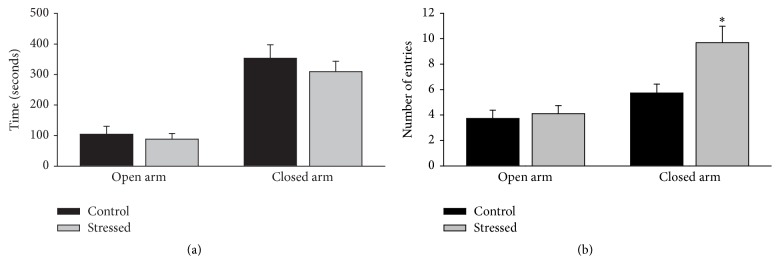
Effect of CMS on male rat's performance in the elevated plus maze. The CMS paradigm did not affect the time spent in the open or closed arms (a), but it increased the number of entries into the closed arms (b), suggesting an anxiogenic effect of CMS. Data are expressed as mean ± SEM. Student's *t*-test: ^*∗*^ *p* < 0.05 versus control group.

The performance of stressed (*n* = 13, gray bars) or unstressed (*n* = 13, black bars) male rats on the elevated plus maze test is shown in Figure 4. The time spent in the open or closed arm (Figure 4(a)) did not present significant differences determined by CMS (*p* = 0.606, *p* = 0.445, resp.). Similarly, the number of entries into the open arm (Figure 4(b)) did not show such difference (*p* = 0.667); in contrast, the statistical analysis of the number of entries into the closed arm indicated that the stressed group showed more entrances than the unstressed group (*p* = 0.010).

### 3.4. Defensive Burying Behavior

**Figure 5:**
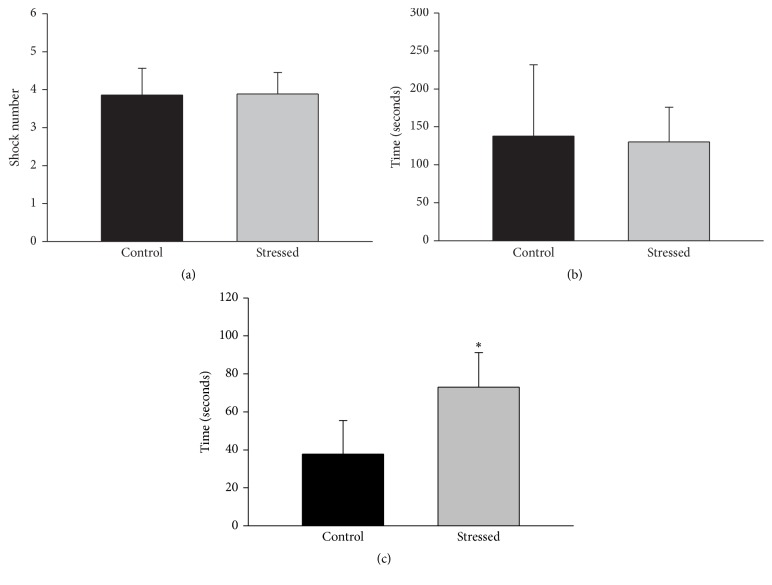
Effect of CMS on the performance of male rats in the defensive burying behavior test. CMS did not affect the number of shocks (a) or burying behavior latency (b); however, it increased the cumulative burying behavior (c), suggesting an anxiogenic effect of CMS. Data are expressed as mean ± SEM. Student's *t*-test: ^*∗*^ *p* < 0.05 versus control group.

Animals that did not receive a shock (2 stressed and 4 control rats) throughout the test were omitted from the analysis; there was no difference between the proportions of stressed and control animals that did not receive the shock (*p* = 0.645, Fisher exact test).  Figure 5 shows the number of shocks (a), burying behavior latency (b), and cumulative burying behavior (c) of rats previously exposed (*n* = 11, black bars) or nonexposed (*n* = 9, gray bars) to CMS and evaluated in the defensive burying behavior test. Data analysis did not indicate differences determined by stress in the shock number or the burying latency (*p* = 0.419 and *p* = 0.818, resp.); in contrast, this analysis showed that the cumulative burying behavior was higher in the stressed group compared to the unstressed one (*p* = 0.035).

### 3.5. Behavioral Despair in the FST

**Figure 6:**
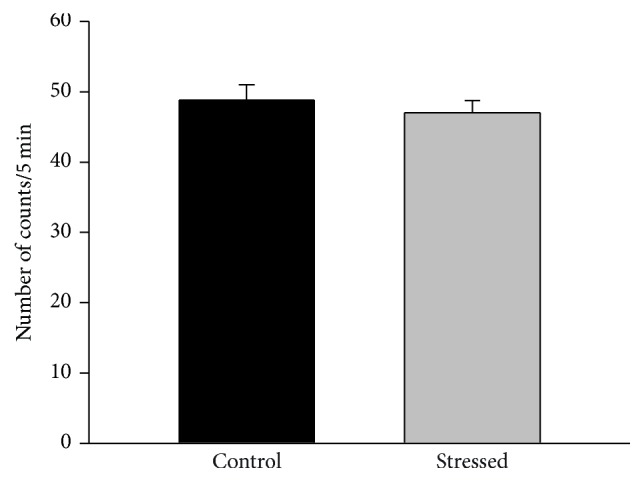
Effect of CMS on the behavioral despair in the FST. There were no significant differences between the control and stressed group in the immobility behavior. Data are expressed as mean ± SEM.

Figure 6 illustrates the immobility behavior in the FST of rats previously exposed to 4 weeks of CMS (*n* = 13, black bar) or those who were kept unstressed (*n* = 13, gray bar). Statistical analysis of these data indicated no significant differences between the control and stressed group (*p* = 0.971).

### 3.6. Corticosterone Serum Levels

Corticosterone serum levels were 242.21 ± 45.56 ng/mL (*n* = 6) for control rats and 197.35 ± 17.14 ng/mL (*n* = 7) for the animals exposed to CMS; statistical analysis did not show significant differences (*p* = 0.349, *t*-test).

## 4. Discussion

### 4.1. CMS as a Paradigm of Depressive-Like State

CMS paradigm is a highly validated animal model that uses stressors of moderate intensity (to give a realistic analogue of the stressor found in everyday life) to generate anhedonia in rodents [7, 8]; this state is indicated by a reduction of the intake of a sucrose solution, which is found to be palatable for most rats. The sensitivity of this parameter to detect anhedonia increases when the animals are deprived of food and water for several hours (14 to 20) before the sucrose solution intake test; this deprivation reduces the within-group variability, resulting in a wider difference between stressed and control animals with respect to nondeprived subjects [16]. In our laboratory, the mild stressors used in the CMS schedule have been effective to induce anhedonia in male rats, showing age differences, insomuch that high and small percentages of middle-aged and young rats express this core sign of depression, respectively [13]. In the present study, we found that young-adult male rats did not reduce the sucrose consumption or sucrose preference when they were exposed to the CMS paradigm during a period of three weeks, which agrees with our previous study [13]. The lack of difference in the anhedonic state between stressed and nonstressed rats cannot be explained by differences in conditions of housing or handling, since they were the same for both groups along the experiment (except for the stressors), including the periods of deprivation of food and water before the sucrose consumption test.

In the same line, present results could suggest variations in the spectrum of physiological or behavioral responses in rodents after application of chronic stressors. Besides anhedonia, CMS paradigm generates other outcomes related to depression (increased immobility in the FST, potentiation of learned helplessness, decreased male sexual behavior, and decreased REM sleep latency) and other stress-related disorders such as anxiety (for review, see [10]). In the same way, several reports indicate that establishment of high levels of anhedonia by means of a CMS schedule could show variations by factors as (1) intrastrain variation; (2) epigenetic or genetic aspects; (3) the laboratory environment; and (4) adverse or stimulating early life events [28]. For the similitude with the multifactorial etiology of human depression, these observations further support the hypothetical validity of the CMS model.

### 4.2. Depressive-Like Behaviors Induced by CMS

In this study, we found that young-adult male rats did not reduce the sucrose consumption or sucrose preference when exposed to the CMS paradigm during a period of three weeks and could suggest that young male rats are resilient to this kind of stress. The resilience of young animals to the CMS paradigm found in the current study contrasts with the reports showing that rats from the same age and strain, exposed to similar stress schedules, developed anhedonia [7, 29, 30]. However, there are also descriptions that some animals are resistant to a particular CMS schedule [31, 32]; although this proportion is relatively lower, it suggests that intrastrain differences are important regarding stress resilience. According to these authors, the difference in vulnerability to CMS could be determined by variations in factors such as hippocampal neurogenesis or cellular plasticity pathways [31, 32]. Alternatively, it could be argued that 3 weeks of CMS is not adequate or enough to induce anhedonia in the animals; however, a similar stress protocol was shown to be effective to induce this state in young-adult Sprague Dawley rats [33]; furthermore, in our laboratory, this scheme of stressors has effectively induced anhedonia in middle-aged [13, 18] and young-adult [17] Wistar male rats. Additionally, in our experience, the exposure of nonanhedonic young-adult male rats to a longer period of CMS (7 weeks) did not change the threshold of rats to exhibit a hedonic state (unpublished data).

In contrast with results of anhedonia, here we found that the body weight gain of the rats was reduced by the CMS paradigm. These data are in line with a previous study made on Sprague Dawley and Long Evans rats where CMS did not induce anhedonia but reduced rats' body weight [34]. It has been proposed that body weight changes indicate male rats' vulnerability to the stress since they reflect the overall impact of a chronic stressful situation [35, 36]. This sign may be interpreted as a manifestation of a depressive-like state in rats since reduction of body weight is considered a symptom of the human disorder, mainly in comorbidity with anxiety disorders [5].

Restriction of body weight in rats exposed to chronic stressors is a physiologic correlate evoked by changes in a central level. Stress response is mediated by a main secretagogue, the 41-aminoacid peptide corticotrophin releasing factor (CRF), which is synthesized in neurons of hypothalamic or extrahypothalamic nuclei (i.e., the paraventricular nucleus and central amygdala, resp.). CFR interacts with two main receptors (CRF1 and CRF2), which are differentially expressed in distinct brain structures and peripheral tissues [37]. A huge number of studies demonstrate that central administration of CRF replicates signs of depression and anxiety in rodents, such as reduced exploration, increased despair, decreased appetite, disrupted sleep, and reduced gain of body weight [38–42]. Furthermore, experiments with drugs that selectively antagonize the CRF1 receptors suggest that these play an important role in the initiation of events that lead to reduction of body weight in rodents without stress exposure (i.e., poor food intake) [43] as well as in downregulation of body weight of animals under CMS [44]. Results shown here suggest that the vulnerability to CMS of some young-adult males seems to be expressed by different signs associated with the depressive spectrum and is not limited to anhedonia.

Given the contrasting results described above and considering that CMS is able to generate behavioral despair in the FST (for review, see [10]), another animal model of depression, we decided to evaluate whether this behavior would better indicate the vulnerability of young rats to the CMS paradigm. Results from the FST indicated that chronic stress did not increase the behavioral despair, suggesting again that the young-male rats are resilient to CMS. This result contrasts with studies that describe an increase of immobility behavior of young animals in the FST after previous exposure to a CMS schedule [19, 20]; however, the current study is in agreement with other reports where CMS did not increase behavioral despair of young-adult rats in the FST [45–47]. A putative explanation for the inconsistency of result among the effect of CMS on FST could be related to variations in the intensity, unpredictability, or duration of stressors used in the stress regime; also, the rat strain used could be a factor of variability. In the current study, immobility levels presented by the male rats could contribute to the lack of effect of CMS on FST; as shown in Figure 6, control male rats presented very high levels of immobility (~50 counts from a total of 60); this characteristic importantly reduces the dynamic range to observe putative stress-related magnification of the depressive-like behavior (an increase of immobility levels) in the animals; thus, it is difficult to detect the impact of CMS on behavioral despair. This effect could be related to the large period (around 2 months) of animal isolation required by the CMS schedule, since this condition alters the animals' performance in the FST [48]; this hypothesis remains to be proved.

It also might be thought that the animals required more than three weeks of CMS exposure to develop high levels of behavioral despair in the FST; however, the exposure of 3 to 4 weeks of CMS has been shown to be enough to effectively increase immobility in the FST [19, 20]. Thus, the discrepancies in the expression of depressive-like behaviors may be determined by differences in stressors intensity and animals' ability to cope with them.

Since anxiety is another psychiatric disease where the stressful events play an important etiologic role, we decided to evaluate the impact of this kind of stress on rodent's anxious-like behaviors.

### 4.3. Anxious-Like Behaviors Induced by CMS

In this study, we evaluated anxious-like behaviors using two tests sensitive to previous stress exposure: elevated plus maze [21] and defensive burying behavior test [22]. In the elevated plus maze test, we found that CMS only increased the number of entries to the closed arms, suggesting that stressed animals have an increased preference for the closed spaces which is considered an anxious-like behavior [23, 24]. In line, although the stressed animal did not avoid the electrode (no differences in the shock number) in the defensive burying test, these animals presented higher cumulative burying behavior with respect to the nonstressed animals; this result indicates that CMS induces an increased aversion for the noxious stimulus in the male rats.

These data together indicate that CMS induced an anxiety-like state in young-adult animals that did not develop anhedonia (as indicated by their sucrose preference; see Figure 2(c)). These results are in line with previous reports [11, 12] where CMS increased anxious-like behaviors in rodents evaluated in several tests. The anxiogenic effect of CMS on young male rats indicates that they are not resilient to this kind of stressor, as previously reported [13]; instead, it appears that young animals' vulnerability to CMS is not reflected as the core symptoms of depression. The anxiogenic-like effects of CMS found in the current study contrast with the study of Kompagne et al. [19] who found ambiguous effects of this paradigm on anxious-like behaviors in Wistar rats (CMS induced social avoidance and increased grooming but acted as if it was anxiolytic in the elevated plus maze). Interestingly, in the study referred to, a clear effect of CMS on depressive-like behaviors was observed. In line, Karson et al. [49] found that Wistar young-adult male rats exposed to the CMS paradigm exhibited depressive-like behavior when evaluated in the sucrose preference test and the FST; however, the animals did not develop behavioral alterations related to anxiety in the elevated plus maze test. Compared with the current CMS schedule, Karson's protocol included periods of restraint (4 h), level shaking (10 min), and nip tail (10 min); meanwhile, it did not include stroboscopic light, white noise, or deprivation of food or water. Thus, all these data suggest that CMS induces either anxiety or depressive-like behaviors in young male rats; this differential effect could be determined by the intensity or duration of the stressors employed in the CMS schedule. This idea is supported by a clinical report suggesting that the nature of the stressor is important in the development of depression or anxiety [50].

It is known that the stressor and the ability to effectively deal with it contribute to the development of anxious and depressive symptoms [3, 4]. This relationship conduces to the coexistence of anxiety and depression symptomatology in the same patients; this fact has led authors to propose a continuum model, from anxiety syndromes to mild, moderate, and severe depression. However, an alternative hypothesis that needs to be explored is whether the comorbidity of anxiety and depression symptomatology is due to linked neurobiological abnormalities that are nevertheless distinct in terms of whether they mediate the anxiety or the depressive symptoms [51]. In this respect, the current study suggests that CMS paradigm could be a useful tool to selectively study neurobiological basis of anxiety and depression. Thus, it will be interesting to study different CMS schedules varied in duration, intensity, or number of stressors per day in order to differentially generate anxious- or depressive-like behaviors in young male rats.

### 4.4. Corticosterone Serum Levels

In the current study, we did not find differences between corticosterone levels of stressed and control rats, suggesting habituation of the animals to the stressors employed in our CMS schedule. This result is in agreement with previous data found in our laboratory where seven weeks of CMS induced a reduction of corticosterone serum levels (345.98 ± 47.73 ng/mL, unstressed group, versus 185.41 ± 56.85 ng/mL, stressed group, *n* = 8, *p* < 0.01, unpublished data); however, the failure of CMS to increase corticosterone levels in young adults contrasts with reports indicating that chronic stress induces elevated levels of glucocorticoids [52, 53]. This discrepancy may be explained by variance in intensity of stressors since in those reports the stressors were more severe (e.g., restraint) than the ones used in the current study; in support of this idea, one of the rules of habituation that appear to govern adaptation to a chronic stressor states that “the progressive reduction of the responses to a repeated stimulus is negatively related to its intensity” [54].

Contrasting with the current results, there are also several reports in which young-adult rats exposed to several weeks of CMS showed elevated corticosterone levels compared with the unstressed animals [34, 55–57]; however, it is important to highlight the notion that in these studies there was a significant proportion of anhedonic rats (~70% according to [58]), as indicated by the reduction in sucrose intake, which is different from the one found in the current study, where no animal developed anhedonia. Thus, such dissimilarities may be, at least in part, explained by differences in behavioral phenotype since in the current study the animals expressed anxious-like instead of depressive-like behaviors.

In the current study, the animals exposed to CMS displayed an anxious-like phenotype which was not related to hyperactivity of HPA axis; this result contradicts the diathesis-stress hypothesis for anxiety and depression; however, in some cases, especially after chronic stress, low corticosterone levels are the feature of HPA axis dysfunction [54, 59] that could be related to affective disorders, as in the case of atypical depression in humans [59, 60]. Furthermore, the corticosterone profile found in our stressed animals is in agreement with a recent study [61] that describes a reduction in corticosterone levels of young rats after chronic stress (21 days of social isolation); despite this, the animals displayed anxious- and depressive-like phenotypes in the elevated plus maze and the FST, respectively; these behavioral results were related to alterations in brain plasticity in the stressed animals (as measured by deficiency in some cell adhesion molecules), which are characteristic of depressive/anxious states [62]. Thus, it appears that hyperactivity of HPA axis is not the unique factor related to the etiology of affective disorders; it is becoming clear that hypoactivity of the HPA axis may result from chronic stress exposure and that this effect may be important in stress-related disorders [63, 64].

An additional interpretation for the occurrence of behaviors related to affective and/or anxiety disorders takes into account the action of CRF on limbic brain structures, such as amygdaloid nuclei. Thus, studies show that administration of this peptide directly into basolateral amygdala [39, 65] or its overexpression in central amygdala [66] induced anxious-like behaviors in rodents. In turn, treatment with antagonists of CRF receptors is able to reduce anxiety-like behaviors and expression of CRF mRNA in the central nucleus of amygdala [67] and restore the gain of body mass in animals submitted to chronic stress [43]. Interestingly, some of these effects are independent of adrenals, since corticosterone levels were not directly corelated to increases or decreases in CRF [42, 43, 68]. Accordingly, some authors propose that this dissociation is due to the actions of corticosterone on different neuronal populations: on PVN neurons for blunting the HPA axis activity (via restriction of CRF production) and on neurons of amygdala for stimulating CRF expression and induction of anxiety-like behaviors. Results presented here seem to be in line with the second option; however, further experiments are needed to test this hypothesis.

Present data together indicate that the relationship between stress and affective disorders is not as simple as it appears; thus, to elucidate the impairments induced by chronic stress on central nervous system that could generate these disorders, an evaluation of the different components of HPA axis and their effect on limbic structures is needed.

## 5. Conclusions

The CMS schedule used in this study induced behavioral alterations that suggest anxiety but not the core symptom of depression, anhedonia, in young-adult males. These results indicate that nonanhedonic young male rats are not resilient to this kind of stressor, but rather the alterations are not expressed in the sphere of motivational behaviors. This study highlights the need to use several physiologic measures (such as body weight) and behavioral tests, when evaluating animal's vulnerability to stress. This study also suggests that CMS could be an animal model to study the neurobiology of depression and anxiety.

## Figures and Tables

**Table 1:** Chronic mild stress schedule.

Time (hours)	First Wed.	Thr.	Fri.	Sat.	Sun.	Mon.	Tue.	Every subsequent Wed.
7:00-8:00			SC/CL	WD			O/CL	FD/WD
8:00-9:00			SC/CL	WD			CT/CL	FD/WD
9:00-10:00			CL	WD			CT/CL	FD/WD
10:00-11:00	*Baseline*		SL	WN			CT/CL	*SPT*
11:00-12:00		O	SL	WN			CT/CL	
12:00-13:00		O	SL	WN		SL	CT	
13:00-14:00	WN	O	SL	WN		SL	CT	WN
14:00-15:00	WN	O	SL			SL	FD/WD	WN
15:00-16:00	WN	O/CL				SL	FD/WD	WN
16:00-17:00		SC/CL	WD			SL	FD/WD	
17:00–7:00		SC/CL	WD			O/CL	FD/WD	

WN: white noise (~90 dB); O: overcrowding (2-3 rats per cage); CL: continuous lighting; SC: soiled cage (250 mL water spilled into bedding); SL: stroboscopic light (300 flashes/min); WD: water deprivation; CT: cage tilt (45°); FD: food deprivation; SPT: sucrose preference test.
